# KOJI AWARENESS, a self-rating whole-body movement assessment system, has intersession reliability and comparability to external examiner rating

**DOI:** 10.1371/journal.pone.0308973

**Published:** 2024-08-15

**Authors:** Hiroshi Takasaki, Shunsuke Kanayasu

**Affiliations:** Department of Physical Therapy, Saitama Prefectural University, Koshigaya, Saitama, Japan; Iran University of Medical Sciences, ISLAMIC REPUBLIC OF IRAN

## Abstract

KOJI AWARENESS is a newly developed self-rating whole-body movement assessment system that includes 11 domains and 22 tests. The primary aim of this study was to investigate the intersession reliability of KOJI AWARENESS, and the secondary aim was to determine whether a fixed bias existed between self-rating and external examiner rating. Fifty university students rated their movement ability in two separate sessions; an external examiner also rated the students’ movement ability. Participants were blinded to their scores at the first session as well as the external examiner’s rating scores. The primary analysis included examining the intersession reliability of the total score with intraclass correlation coefficients (ICCs). ICC values were interpreted as follows: insufficient, < .7 and sufficient, ≥ .7. To achieve the secondary aim, Bland–Altman analysis was performed. ICC for the intersession reliability was .86 with a 95% confidence interval (CI) of .77 to .92 and a minimum detectable change (MDC) of 5.15. Bland–Altman analysis revealed fixed bias as the 95% CIs of the mean difference between the two different rating scores (−3.49 to −2.43 and −3.94 to −2.98 in the first and second sessions, respectively) did not include 0 in the data of each session. However, no proportional bias was identified because no statistically significant Pearson’s correlation (P > .05) was noted between the means of the two methods and the mean difference between the two different methods in each session. This study identified that KOJI AWARENESS has sufficient intersession reliability among relatively young and healthy participants. External examiner rating tended to have lower total scores than self-rating; however, the bias was below the MDC and seemed not to be clinically significant.

## Introduction

Maintaining movement ability throughout life is essential for health and wellness. Self-monitoring is necessary to maintain movement ability. In particular, an assessment tool to monitor whole-body movement ability is an efficient self-monitoring strategy. Functional limitations in a specific part of the body can overload other parts and consequently affect whole-body postural control [1]; therefore, to identify problems that are not captured by the functional assessment of individual parts of the body, whole-body movement assessment would be beneficial.

There are several performance-based outcome measures (PBOMs) on whole-body movement assessment, including the Selective Functional Movement Assessment (SFMA) and Functional Movement Screen (FMS). However, these PBOMs require experienced examiners. In 2022, a self-assessment PBOM on whole-body movement ability without instruments was developed and named as KOJI AWARENESS. It incorporates movement tests of 11 components, with individuals marking their own movement score. This assessment is designed to be applied to individuals of all ages, with or without pain or musculoskeletal problems, as long as the instructions are understood. The total score of KOJI AWARENESS is correlated with that of FMS [2], an established PBOM with interexaminer reliability [3]. This finding supports the validity of KOJI AWARENESS. Moreover, there is emerging evidence supporting its usefulness, such as KOJI AWARENESS scores correlated with the degree of pain during training in athletes, and pain during training was also reduced after exercises that improved the KOJI AWARENESS score [4,5].

However, the reliability of this tool has not been fully investigated yet. KOJI AWARENESS is a self-assessment tool; hence, investigating its intersession reliability is a research priority. Furthermore, interexaminer reliability, i.e., reliability between self-rating and examiner rating, may not be a research priority because a correlation between examiner rating movement ability using FMS and self-assessment using KOJI AWARENESS has already been reported [2]. However, there can be a difference between the examiner rating score and the self-rating score.

This study primarily aimed to investigate the intersession reliability of KOJI AWARENESS. The secondary aim was to investigate whether a fixed bias existed between self-rating and external examiner rating.

## Materials and methods

### Design

This study used a test–retest design, with participants’ self-rating scores and examiner’s rating scores from observation being blinded to the participants and examiner throughout the experiment. Further, self-scores at the first session were blinded to the participants and examiner at the second session. The second session was conducted within 1 week on a different day. Before data collection, each participant provided their written informed consent. This study was approved by an institutional research ethics committee (Saitama Prefectural University, No. 23018) and complied with the Declaration of Helsinki.

### Participants and an external examiner

Fifty students were recruited in a local university (Saitama Prefectural University, Japan) from June 5, 2023 to January 17, 2024. Individuals who were unable or contraindicated to perform the movements such as standing up, sitting down, standing on one leg, kneeling, or bending the trunk were not eligible to participate in this study. We asked the participants not to change their exercise habits during the study and excluded those who changed their exercise habits between the first and second measurement weeks due to aggravation of injury or some other reasons. The number of participants was determined according to an adequate criterion in the consensus-based standards for the selection of health measurement instruments (COSMIN) guidelines [6].

The external examiner (SK) was a third year university student studying physical therapy. Prior to this study, he had participated in a level 1 workshop on SFMA to gain skills on movement observation. The examiner participated in this study after having fully understood KOJI AWARENESS by repeatedly watching the video (S1 File).

In addition to basic demographic information of the participants (i.e., age, sex, and body mass index [kg/m^2^] calculated with height and weight), considering future data accumulation, we used the EuroQol 5 Dimension (EQ5D) [7], which is free, multidimensional in content, and easy to use, to generally understand participant’s health status. The International Physical Activity Questionnaire short version (IPAQ) [8,9] was also collected to allow comparisons with future reliability studies in different populations as physical activity level may be associated with movement ability. The EQ5D is a reliable and valid patient-reported outcome measure of health status that includes five items of mobility, self-care, usual activities, pain or discomfort, and anxiety or depression [10]; a higher EQ5D score indicates better health status (0–1) [11]. The IPAQ is a reliable and valid self-reporting questionnaire for assessing average activity level in minutes × Mets [9,12,13]; a higher IPAQ score indicates greater activity level.

### KOJI AWARENESS

KOJI AWARENESS includes the following 11 domains (Table 1), where details are presented in an S1 File in the previous study (https://doi.org/10.1371/journal.pone.0277167.s002) [2]. The total score which ranges from 0 to 50 and indicates an individual’s whole-body movement ability. The movements, notes, and scoring criteria for each movement test (https://doi.org/10.1371/journal.pone.0277167.s003) [2] were presented in a video, with 11 videos of 2–5 minutes each corresponding to the 11 components (S1 File).

**Table 1:** KOJI AWARENESS structure.

Domain	Tests	Domain scores
**(1) Neck mobility**	6 tests (flexion, extension, left and right side bending, and left and right rotation)	0–6 (Score of 0–1 × 6 tests)
**(2) Shoulder mobility**	2 tests (left and right sides)	0–2 (Score of 0–1 × 2 tests)
**(3) Scapular mobility**	2 tests (left and right sides)	0–2 (Score of 0–1 × 2 tests)
**(4) Thoracic spine mobility**	2 test (left and right sides) with 3 grades	0–6 (Score of 0–1 × 2 test×3 grades)
**(5) Upper extremity stability and strength**	1 test with 4 grades	0–4 (Score of 0–1 × 1 test×4 grades)
**(6) Hip mobility**	8 tests (flexion and external rotation of the left and right sides, flexion and internal rotation of the left and right sides, extension and external rotation of the left and right sides, and extension and internal rotation of the left and right sides)	0–8 (Score of 0–1 × 8 tests)
**(7) Hip and spinal mobility**	2 tests (front and back sides) with 3 grades	0–6 (Score of 0–1 × 2 tests × 3 grades)
**(8) Upper and lower extremity mobility and stability**	2 tests (left and right sides)	0–2 (Score of 0–1 × 2 tests)
**(9) Mid-section stability strength**	1 test with 4 grades	0–4 (Score of 0–1 × 1 test×4 grades)
**(10) Lower extremity strength**	2 tests (left and right sides) with 4 grades	0–8 (Score of 0–1 × 2 tests× 4 grades)
**(11) Ankle mobility**	2 tests (left and right sides)	0–2 (Score of 0–1 × 2 tests)

A score of 1 is given when a movement was completely performed in each test and each grade.

No warm-up was allowed before the measurement. For each test and each measurement session, participants watched the video and moved spontaneously, and subsequently marked whether they met the criteria for that test on a sheet with prescored criteria. If participants did not understand a test movement, they were allowed to stop the video and repeat it as many times as they wished. To determine their score, each test was performed once. No instructions or feedback were provided by the external examiner. The examiner moved around freely to observe the participants’ test movements from the best angle. The participants and the external examiner scored simultaneously. To maintain examiner blinding, the above process was conducted under the supervision of the first author, with the two examiners submitting data separately to the first author.

### Analysis

The intersession reliability of the self-rating and examiner-rating scores was examined using probability-adjusted kappa (PABAK) for the 22 tests with binary data (neck mobility; shoulder mobility; scapular mobility; hip mobility; and upper and lower extremity mobility and stability) and probability-adjusted kappa-ordinal scale (PABAK-OS) for the five tests with categorical data (thoracic spine mobility; upper extremity stability and strength; hip and spinal mobility; mid-section stability strength; and lower extremity strength). Moreover, PABAK-OS was also calculated in each domain. For the relative reliability of the total score, intraclass correlation coefficients based on a two-way mixed-effects mode (ICCs_[3,1]_) were calculated based on the ICC guideline [14]. For absolute reliability, minimum detectable changes (MDCs) were calculated using the standard deviations of the values and the ICC values on the basis of the following formulas:

$$SEM=SD\sqrt{1-ICC}$$ (1)

$$MDC=SEM\times 1.96\times \sqrt{2}$$ (2)

Interpretations of the ICC and kappa values were as follows: insufficient, < .7; sufficient, ≥ .7 [15].

Fixed and proportional biases were examined for the total score using the Bland–Altman analysis [16]. Fixed bias was identified when the 95% confidence intervals (CIs) of the mean difference between the two different rating scores ($\bar{d}$) did not include 0. The 95% CIs of the $\bar{d}$ value was calculated using the following formula:

$$\bar{d}\pm t\times \sqrt{\frac{{SD}_{d}}{n}}$$ (3)

where n indicates the number of the participants; SD_d_ indicates the standard deviations of the $\bar{d}$ value; and t represents the degree of freedom of n–1. Proportional bias was identified when a statistically significant Pearson’s correlation (r) was noted in the mean difference between the two different methods ($\bar{d}$) and the mean of the two different methods.

IBM SPSS Statistics for Windows version 28.0 was used for all statistical analyses, except PABAK-OS, which was performed online [17]. The significance level was set at 5%.

## Results

None of the participants changed their exercise routine between the first and second weeks of measurement, and all were included in the analysis. No participants had any events influencing body movements, including injury or aggravation of pain, during the measurement and between the two assessments. The characteristics of the 50 participants are summarized in Table 2. Nine participants (18%) reported some pain in the pain/discomfort section of the EQ5D, but the degree of pain was slight for all of them, and none of them reported any problems with self-care or their usual activities.

**Table 2:** Summary of characteristics of the 50 participants.

**Age, mean (SD), y**	20.6 (1.7)
**Sex, No. (%)**	Women, 27 (44.0%) Men, 23 (46.0%)
**EuroQol 5 Dimension, mean (SD), 0–1**	0.95 (0.09)
**Body Mass Index, mean (SD), kg/m** ^**2**^	20.7 (2.0)
**International Physical Activity Questionnaire short version, mean (SD), minutes × Mets**	2324.4 (1939.0)

**Fig 1:**
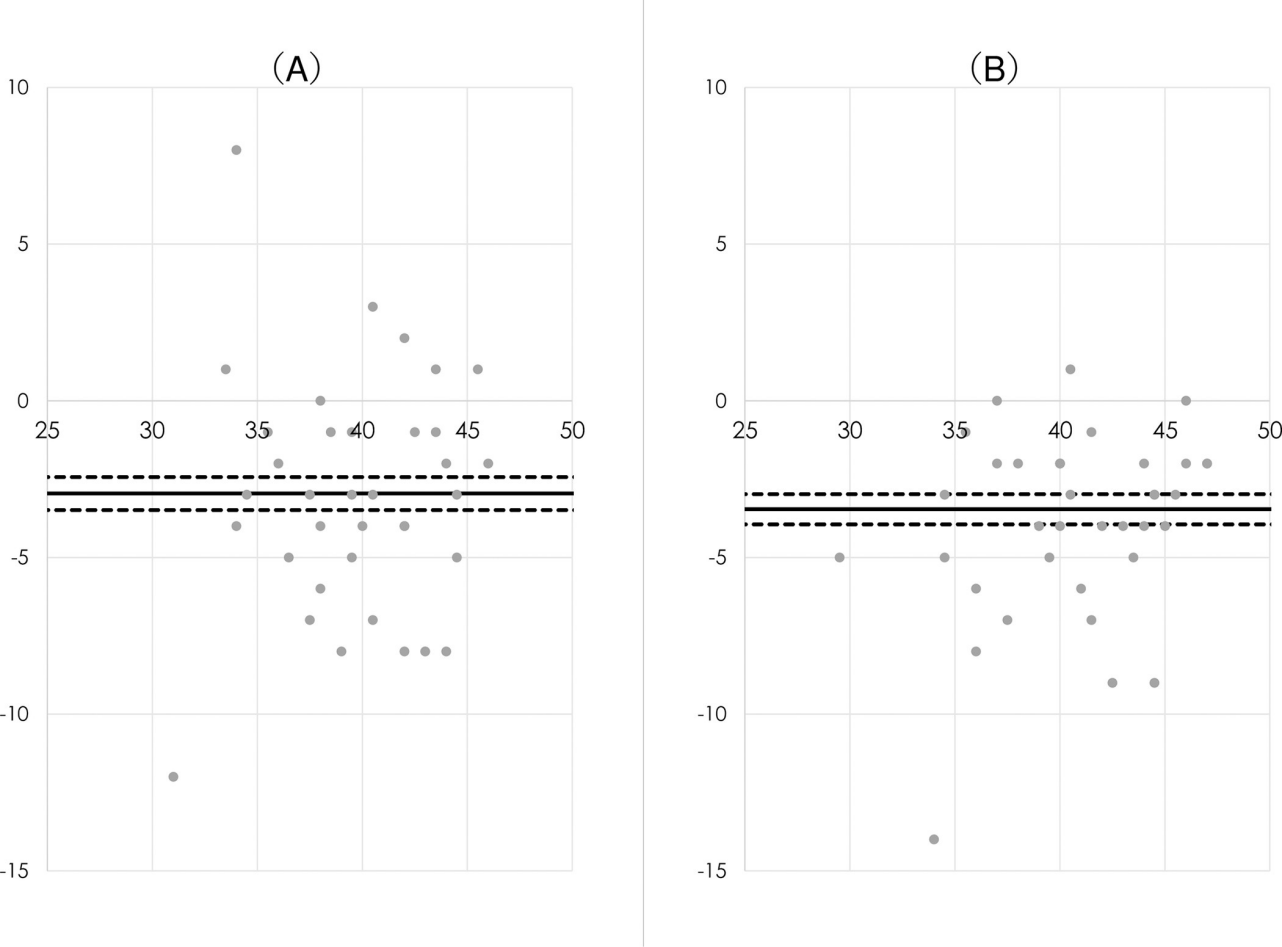
Bland-Altman plots for comparison of the external examiner rating than self-rating. X axis: Mean of the two different rating scores. Y axis: Difference from the external examiner rating scores to self-rating scores. Solid line: Mean difference between the two different rating scores ($\bar{d}$). Dash lines: Upper and lower 95% confidence intervals of the $\bar{d}$ value. (A) 1^st^ session. (B) 2^nd^ session.

The PABAK values for interexaminer and intersession reliabilities in each test are presented in Table 3. The reliability in most tests (85%: 23/156 tests) was interpreted as sufficient. The relative and absolute reliabilities are summarized in Table 4, wherein the interexaminer and intersession reliabilities of the total score were interpreted as sufficient. The mean (SD) of the KOJI AWARENESS total scores were 38.54 (4.65) for examiner rating, and 41.50 (4.87) for self-rating at the first session; and 39.20 (5.42) for examiner rating, and 42.66 (5.03) for self-rating at the second session, respectively. With respect to the overall score, there were neither ceiling nor floor effects that exceeded 15% of the response frequency [18–20]. Bland–Altman plots are presented in Fig 1, where the 95% CIs of the $\bar{d}$ value did not include 0. Thus, fixed bias was identified in each session, indicating lower total scores in external examiner rating than those in self-rating. However, no proportional bias was identified (*r* = −.07 [95% CIs: −.34 to .21], P = .639 in the first session; *r* = .14 [−.14 to .40], P = .329 in the second session).

**Table 3:** Probability adjusted kappa values for interexaminer and intersession reliability in each test.

Test	Interexaminer		Intersession	
	1st session	2nd session	External examiner rating	Self-rating
**Neck mobility domain**	0.17 (0.07 to 0.26)	0.25 (0.16 to 0.35)	0.39 (0.30 to 0.49)	0.46 (0.37 to 0.56)
**Flexion**	0.52 (-0.29 to 1.33)*	0.52 (0.29 to 0.75)*	0.64 (0.36 to 0.92)*	0.80 (0.54 to 1.06)*
**Extension**	0.76 (0.51 to 1.01)*	0.80 (0.56 to 1.04)*	0.68 (0.41 to 0.95)*	0.88 (0.62 to 1.14)*
**Left side bending**	0.76 (0.51 to 1.01)*	0.72 (0.44 to 1.00)*	0.56 (0.28 to 0.84)*	0.76 (0.49 to 1.03)*
**Right side bending**	0.72 (0.44 to 1.00)*	0.68 (0.41 to 0.95)*	0.64 (0.36 to 0.92)*	0.60 (0.32 to 0.88)*
**Left rotation**	0.44 (0.25 to 0.63)	0.12 (-0.07 to 0.31)	0.68 (0.42 to 0.94)*	0.44 (0.18 to 0.70)*
**Right rotation**	0.40 (0.17 to 0.63)	0.32 (0.09 to 0.55)	0.72 (0.44 to 1.00)*	0.32 (0.04 to 0.60)*
**Shoulder mobility domain**	0.94 (0.81 to 1.07)*	1.00 (0.87 to 1.13)*	0.97 (0.84 to 1.10)*	0.97 (0.84 to 1.10)*
**Left**	0.96 (0.70 to 1.22)*	1.00 (0.72 to 1.28)*	0.96 (0.70 to 1.22)*	1.00 (0.72 to 1.28)*
**Right**	0.96 (0.68 to 1.24)*	1.00 (0.72 to 1.28)*	1.00 (0.72 to 1.28)*	0.96 (0.68 to 1.24)*
**Scapula mobility domain**	0.25 (0.12 to 0.38)	0.22 (0.09 to 0.35)	0.94 (0.81 to 1.07)*	0.70 (0.57 to 0.83)*
**Left**	0.20 (0.08 to 0.32)	0.12 (-0.01 to 0.25)	0.96 (0.69 to 1.23)*	0.72 (0.44 to 1.00)*
**Right**	0.12 (0.01 to 0.23)	0.00 (-0.10 to 0.10)	0.92 (0.64 to 1.20)*	0.72 (0.44 to 1.00)*
**Thoracic spine mobility domain**	0.63 (0.53 to 0.72)*	0.79 (0.69 to 0.89)*	0.70 (0.60 to 0.79)*	0.70 (0.60 to 0.79)*
**Left**	0.65 (0.53 to 0.77)*	0.81 (0.69 to 0.93)*	0.73 (0.61 to 0.85)*	0.79 (0.67 to 0.91)*
**Right**	0.68 (0.56 to 0.80)*	0.81 (0.69 to 0.93)*	0.68 (0.56 to 0.80)*	0.71 (0.59 to 0.83)*
**Upper extremity stability and strength domain**	0.93 (0.81 to 1.04)*	0.93 (0.81 to 1.04)*	0.98 (0.87 to 1.09)*	0.95 (0.84 to 1.06)*
**Hip mobility domain**	0.21 (0.13 to 0.30)	0.35 (0.26 to 0.43)	0.28 (0.19 to 0.37)	0.21 (0.13 to 0.30)
**Left, flexion and external rotation**	0.36 (0.09 to 0.63)	0.48 (0.24 to 0.72)*	0.52 (0.24 to 0.80)*	0.56 (0.31 to 0.81)*
**Right, flexion and external rotation**	0.44 (0.17 to 0.71)*	0.56 (0.30 to 0.82)*	0.52 (0.26 to 0.78)*	0.48 (0.22 to 0.74)*
**Left, flexion and internal rotation**	0.72 (0.45 to 0.99)*	0.88 (0.60 to 1.16)*	0.68 (0.41 to 0.95)*	0.52 (0.26 to 0.78)*
**Right, flexion and internal rotation**	0.56 (0.28 to 0.84)*	0.64 (0.37 to 0.91)*	0.52 (0.25 to 0.79)*	0.68 (0.40 to 0.96)*
**Left, extension and external rotation**	0.56 (0.29 to 0.83)*	0.60 (0.33 to 0.87)*	0.56 (0.29 to 0.83)*	0.52 (0.25 to 0.79)*
**Right, extension and external rotation**	0.44 (0.18 to 0.70)*	0.68 (0.41 to 0.95)*	0.32 (0.05 to 0.59)	0.40 (0.13 to 0.67)
**Left, extension and internal rotation**	0.60 (0.33 to 0.87)*	0.64 (0.38 to 0.90)*	0.52 (0.24 to 0.80)*	0.64 (0.36 to 0.92)*
**Left, extension and internal rotation**	0.44 (0.19 to 0.69)	0.36 (0.12 to 0.60)	0.44 (0.16 to 0.72)*	0.76 (0.48 to 1.04)*
**Hip and spinal mobility domain**	0.88 (0.79 to 0.98)*	0.93 (0.83 to 1.03)*	0.93 (0.83 to 1.03)*	0.81 (0.72 to 0.91)*
**Front**	0.87 (0.75 to 0.99)*	0.92 (0.80 to 1.04)*	0.73 (0.61 to 0.85)*	0.89 (0.77 to 1.01)*
**Back**	0.97 (0.85 to 1.09)*	1.00 (0.88 to 1.12)*	0.84 (0.72 to 0.96)*	0.87 (0.75 to 0.99)*
**Upper and lower extremity mobility and stability domain**	0.85 (0.72 to 0.98)*	0.91 (0.78 to 1.04)*	0.82 (0.69 to 0.95)*	0.79 (0.66 to 0.92)*
**Left**	0.88 (0.60 to 1.16)*	0.96 (0.68 to 1.24)*	0.84 (0.57 to 1.11)*	0.76 (0.49 to 1.03)*
**Right**	0.92 (0.64 to 1.20)*	0.92 (0.65 to 1.19)*	0.84 (0.56 to 1.12)*	0.84 (0.57 to 1.11)*
**Mid-section stability strength domain**	0.95 (0.84 to 1.06)*	0.98 (0.86 to 1.09)*	0.98 (0.86 to 1.09)*	0.95 (0.84 to 1.06)*
**Lower extremity strength domain**	0.87 (0.78 to 0.95)*	1.00 (0.91 to 1.09)*	0.96 (0.87 to 1.04)*	0.96 (0.87 to 1.04)*
**Left**	0.95 (0.84 to 1.06)*	0.95 (0.84 to 1.06)*	0.95 (0.84 to 1.06)*	0.95 (0.84 to 1.06)*
**Right**	0.90 (0.79 to 1.01)*	1.00 (0.89 to 1.11)*	0.98 (0.86 to 1.09)*	0.93 (0.81 to 1.04)*
**Ankle mobility domain**	0.94 (0.81 to 1.07)*	0.97 (0.84 to 1.10)*	0.97 (0.84 to 1.10)*	0.94 (0.81 to 1.07)*
**Left**	1.00 (0.72 to 1.28)*	0.96 (0.69 to 1.23)*	0.96 (0.69 to 1.23)*	1.00 (0.72 to 1.28)*
**Right**	0.92 (0.68 to 1.16)*	0.96 (0.70 to 1.22)*	0.96(0.70 to 1.22)*	0.92 (0.68 to 1.16)*

*Kappa values of ≥ .7, indicating sufficient reliability.

**Table 4:** Results of interexaminer and intersession reliability in total scores.

Measures	Interexaminer		Intersession	
	1st session	2nd session	External examiner rating	Self-rating
**ICC (95% CIs)**	.74 (.58 to .84)	.85 (.75 to .91)	.88 (.80 to .93)	.86 (.77 to .92)
**SEM**	2.53	2.13	1.75	1.86
**MDC**	7.02	5.89	4.84	5.15

Abbreviations: ICC, intra-class correlation coefficients with case 3; CIs, confidence intervals; SEM, standard error of measurement; MDC, minimum detectable change.

## Discussion

This study evaluated the test–retest reliability of KOJI AWARENESS between external examiner rating scores and self-rating scores. Murohushi et al. [4] reported that the self-rating total score of the KOJI AWARENESS had acceptable test-retest reliability, but the details of the method had not been clarified. To the best of the authors’ knowledge, this study is the first detained verification of the reliability of KOJI AWARENESS.

For the total KOJI AWARENESS score, the ICC exceeded .7 for both the external examiner rating and self-rating scores. Furthermore, both MDCs were comparable at approximately 10% of the total score. Previous studies interpreted an MDC of < 10% as excellent reliability and an MDC of 10%–30% as acceptable reliability [21,22]. Therefore, the total score of the KOJI AWARENESS is considered reliable enough for both the external examiner rating and self-rating scores.

This study revealed a fixed bias in the total score of the KOJI AWARENESS between the external examiner and self-rating scores. For example, a movement that the participant perceived as not moving might be moving from the perspective of an external examiner, or a movement that the participant perceived as being completed up to a certain angle might not be sufficient from the perspective of an external examiner. Thus, the external examiner scored more strictly than the self-rating scores. However, the 95% CIs of the $\bar{d}$ value were below the MDC of test–retest reliability for both the first and second sessions. Therefore, the overall KOJI AWARENESS score, whether scored by an external examiner or by self-rating, is not expected to affect clinical interpretation. The criterion-related validity of the KOJI AWARENESS is already known as its self-rating total score has been confirmed to correlate with the total score of the FMS [2]. Therefore, the findings of this study reveal the clinical utility of KOJI AWARENESS by allowing the assessment of one’s general overview of his or her whole-body movement ability with a total score of KOJI AWARENESS.

A detailed analysis of the individual tests and domain scores showed that the neck and hip mobility domains had insufficient reliability. Furthermore, the interexaminer reliability was insufficient for both the first and second sessions in the scapula mobility domain, despite sufficient intersession reliability for the external examiner rating and self-rating. Thus, these three tests are considered to present with discrepancies in judgment between external examiner rating and self-rating. These tests may need additional or revised KOJI AWARENESS scoring criteria and/or instructions. In particular, as the sensation that the cervical spine or trunk is immobile or not vertical may be altered by symptoms [23,24], it would be prudent to consider how rigorous this criterion should be.

## Limitations

There are several limitations in this study. The first limitation relates to the selection of the participants. The majority of participants in this study were healthy university students in their early twenties. This study identified a trend that the external examiner scored more strictly than the self-rating scores, which may have affected the results of this study if the target population had been the general public, who had less interest in and understanding of movement than the university students who participated in this study. This study did not include in-depth interviews about pain. Although some of the participants in this study experienced pain by chance, the authors believe that it was not a limitation that seriously affected the results of this study because the degree of pain was low and there was no subjective functional limitation. On the other hand, pain with certain intensity can influence movement patterns [25–27]. Certain interventions can also change movement patterns [4,25]. Therefore, the test–retest reliability of the KOJI AWARENESS may be lower in those who have substantial changes in pain status and those who receive certain interventions to correct movement patters. Moreover, the reliability may be lower than that observed in the present study in individuals with suspected cognitive decline. To widely disseminate KOJI AWARENESS to the public in the future, verifying the test–retest reliability for individuals with various characteristics is necessary, and this study is valuable in providing a foundation for future considerations of such a study. The second limitation of the study was that there was only one external examiner and he was an undergraduate student studying physical therapy. Although this student had taken the SFMA Level 1 training and may have been more capable of seeing movement than other students, the results may have been different from this study if several movement assessment experts had served as external examiners. However, the clinically important aspect of this study is the test-retest reliability of the self-ratings, and the authors believe that the limitations of this study do not diminish its value. The third limitation is that we did not collect information on participants’ dominant hand and dominant foot. In this study, test-retest reliability in each test was analyzed separately for left and right sides, but further findings could have been obtained by analyzing the results by dominance sides.

## Conclusion

This study revealed that KOJI AWARENESS has sufficient intersession reliability among relatively young and healthy participants. The external examiner rating tended to show lower total scores than self-rating; however, the bias seemed not clinically significant.

## Supporting information

S1 File KOJI AWARENESS videos. (DOCX)

S2 File Anonymous data. (XLSX)
