# Supplementary material for: KOJI AWARENESS, a self-rating whole-body movement assessment system, has intersession reliability and comparability to external examiner rating
Source: PLoS One. 2024 Aug 15;19(8):e0308973. doi: 10.1371/journal.pone.0308973 (PMC11326541; doi:10.1371/journal.pone.0308973)
Supplement: S1 File — (DOCX) [file pone.0308973.s001.docx]

**S1 File.** **KOJI AWARENESS videos**

Neck mobility

<https://www.youtube.com/watch?v=4-YHy0wMT2w>

Shoulder mobility

<https://www.youtube.com/watch?v=28rGP7_Em0o>

Scapular mobility

<https://www.youtube.com/watch?v=h4HQXcCLheg>

Thoracic spine mobility

<https://www.youtube.com/watch?v=1XfvF2KuKiY>

Upper extremity stability and strength

<https://www.youtube.com/watch?v=dFKL0Q5tUDw>

Hip mobility

<https://www.youtube.com/watch?v=2fAcERo4nlQ>

Hip and spinal mobility

<https://www.youtube.com/watch?v=wNSE6O2TEtg>

Upper and lower extremity mobility and stability

<https://www.youtube.com/watch?v=WaTpKF_8VfM>

Mid-section stability strength

<https://www.youtube.com/watch?v=eFRDnaWmecs>

Lower extremity strength

<https://www.youtube.com/watch?v=JYpm1e1gd2U>

Ankle mobility

<https://www.youtube.com/watch?v=eK3I9CmWEhw>
